# ERAS Society Recommendations for Improving Perioperative Care in Low- and Middle-Income Countries Through Implementation of Existing Tools and Programs: An Urgent Need for the Surgical Safety Checklist and Enhanced Recovery After Surgery

**DOI:** 10.1007/s00268-021-06279-x

**Published:** 2021-08-28

**Authors:** Ravi Oodit, Bruce Biccard, Gregg Nelson, Olle Ljungqvist, Mary E. Brindle

**Affiliations:** 1 Global Surgery Unit, Department of Surgery, University of Cape Town, Anzio Road, Observatory, Cape Town, 7935 South Africa; 2 Department of Anesthesia and Perioperative Medicine, University of Cape Town, Anzio Road, Observatory, Cape Town, 7935 South Africa; 3 Departments of Obstetrics and Gynecology and Oncology, Cumming School of Medicine, University of Calgary, Canada 1331 29 Street NW, Calgary, AB T2N 4N2 Canada; 4 Department of Surgery, School of Medical Sciences, Örebro University, Örebro, Sweden; 5 Department of Surgery, Örebro University Hospital, 701 85 Örebro, Sweden; 6 Department of Surgery, Cumming School of Medicine, University of Calgary, Calgary, AB Canada; 7 Canada and Safe Surgery and Safe Systems, Ariadne Labs, Harvard TH Chan School of Public Health, Brigham and Women’s Hospital28 Oki Drive, Calgary, AB T3B 6A8 Canada

The Lancet Commission and Global Surgery Foundation in 2015 highlighted the need for access to safe and affordable surgical and anesthetic care in low- and middle-income countries (LMICs) [1]. Patients that do have access to care in LMICs, however, have a higher risk of complications and mortality than in high-income countries (HICs). Ninety-six percent of all perioperative deaths worldwide occur in LMICs, and the economic impact of this is a staggering 2.6% of the combined gross domestic product of LMICs [1]. Although it is a common belief that the greatest contributors to adverse outcomes in LMICs are poor access to care and late presentation, deficits in the quality of accessible care are a substantial concern.

Following the Lancet Commission and the World Health Assembly Resolution 68.15, all member countries committed to developing a National, Surgical, Obstetric and Anaesthesia Plan (NSOAP) to assist in improving access to safe surgery and anesthesia [1]. The missing link in the NSOAP strategy is support for the implementation of standardized, evidence-based perioperative care guidelines and tools to measure guideline compliance and outcomes. This is crucial not only because of the need to improve perioperative care but as access to safe surgery and anesthesia improves, there is likely to be increased patient volume and pressure on the healthcare system to provide quality surgical care. A new set of tools need not be developed to improve perioperative care in LMICs. These tools already exist with evidence for their effectiveness. The Surgical Safety Checklist (SSC) and Enhanced Recovery After Surgery (ERAS) Program are two examples [2, 3]. Barriers to acceptance, adoption, and implementation of existing tools present the greatest hurdles that must be overcome to improve perioperative outcomes in LMICs.

The SSC is a communication tool used by the surgical team to confirm that appropriate actions are taken in the perioperative period to maintain patient safety. At the same time, the three pause points within the checklist include conversation prompts to ensure there is a shared understanding between the surgical team members. The SSC was designed to optimize its effectiveness in LMICs with a focus on influencing globally relevant outcomes using recommendations that are applicable and supported by the resources in LMICs. As a result, the use of the SSC has been shown to significantly reduce perioperative morbidity and mortality in LMICs as well as in HIC settings, and its impact may be larger when implemented well in LMICs [2].

Despite evidence of effectiveness, the acceptance and adoption of the SSC remain poor in LMICs with ranges between 20 and 40% when compared with facilities in HIC where rates of adoption range between 80 and 95% [4]. The reasons for this failed penetrance relate to a lack of resources and infrastructure for initial and ongoing implementation and audits and surgical hierarchies that may not support aspects of the SSC, such as encouraging all members of the team to vocalize concerns if they exist. The barriers to successful implementation are further exacerbated by checklist fatigue and similar factors that also lead to decreased meaningful use in HICs. The need for improved implementation of the SSC in LMICs has been recognized by global health organizations. With this increased focus on quality and safety initiatives and implementation, it is time to consider other strategies for improvement.

ERAS is another tool that has the potential to benefit LMICs with strategies that have demonstrated benefits across a variety of settings and clinical outcomes [3]. The ERAS program is based on implementation of evidence-based clinical practice guidelines performed by a multidisciplinary perioperative team, using tools to monitor and evaluate compliance to the guidelines and patient outcomes concurrently. Randomized trials of ERAS-based care vs traditional care conducted in HICs have shown a significant reduction in length of stay (20–40%) and complications (20–30%). Cost studies of ERAS have demonstrated a return-on-investment ratio up to 7.3 (i.e., a savings of $7.3 for every $1 invested), showing that ERAS is value-based surgery [3].

There are few established ERAS programs in LMICs, however, data from these centers demonstrate similar benefits to HICs [5]. Whether these benefits can be achieved at scale remains unknown, and the crux of the issue relates to how ERAS is applied in tertiary-university centers in LMICs compared to the district and regional levels. ERAS guidelines in their current format are specialty-specific, predominantly for elective procedures, and thus likely to be easily implemented in tertiary-university LMIC hospitals, which have similar subspecialty units. The implementation in these units will have the added benefit of facilitating the teaching and training of all perioperative team members.

The greatest unmet surgical and anesthetic need is, however, at the district and regional level in LMICs [1]. Unlike tertiary hospitals, surgery in these centers is often performed on an emergency basis by surgeons with no sub-specialty training. To address this gap, the ERAS® Society, in partnership with the World Bank and perioperative leaders in LMICs, has undertaken the development of a generic perioperative ERAS® Society guideline for elective and emergency surgery. This approach will integrate the SSC and be applied to patients undergoing a variety of operations including general and obstetrical surgery. These practices will focus on key ERAS measures such as patient education/engagement, avoidance of opioids and prolonged fasting, early mobilization, and early feeding. In addition to these guidelines, the ERAS® Society and World Bank are developing a tailored implementation program and monitoring tool to assess guideline compliance and patient outcomes specifically targeted to LMICs.

ERAS and the SSC share a similar quality that makes them well-suited for adoption in poorly resourced settings—that is their adaptability. Both tools are designed to be tailored to suit the context in which they will be adopted. Combining the NSOAP strategy with existing tools such as SSC and ERAS have the potential to provide a platform to improve the quality of surgical care in LMICs with improved patient outcomes and service efficiency, at scale, rapidly and make a significant contribution to addressing the unmet surgical and anesthetic need in LMICs.
